# Transition from Laparoscopic to Robot-Assisted Partial Nephrectomy: Perioperative Outcomes During an Institutional Transition in a High-Volume European Centre

**DOI:** 10.3390/jcm15124746

**Published:** 2026-06-18

**Authors:** Jure Bizjak, Andraž Kondža, Kosta Cerović, Milan Medved, Simon Hawlina

**Affiliations:** 1 Department of Urology, University Medical Centre Ljubljana, 1000 Ljubljana, Slovenia; jure.bizjak@kclj.si (J.B.); andraz.kondza@kclj.si (A.K.); kosta.cerovic@kclj.si (K.C.); milan.medved@kclj.si (M.M.); 2 Faculty of Medicine, University of Ljubljana, 1000 Ljubljana, Slovenia

**Keywords:** robot-assisted partial nephrectomy, laparoscopic partial nephrectomy, renal cell carcinoma, nephron-sparing surgery, minimally invasive surgery, warm ischaemia time, surgical outcomes, complications, renal function, enucleation

## Abstract

**Background/Objectives:** Robot-assisted partial nephrectomy (RAPN) has increasingly replaced laparoscopic partial nephrectomy (LPN) in the management of localized renal tumours. This study aimed to evaluate perioperative, functional and surgical margin outcomes during an institutional transition from LPN to RAPN in a high-volume centre. **Methods:** We performed a retrospective single-centre analysis of 100 consecutive patients undergoing minimally invasive partial nephrectomy. The last 50 LPN cases (August 2014–May 2018) were compared with the first 50 RAPN cases (June 2018–February 2020). Baseline characteristics, perioperative outcomes, early functional parameters and surgical margin status were analysed. Complications were classified according to the Clavien–Dindo system. **Results:** Tumours treated in the RAPN group were significantly larger (3.4 vs. 2.5 cm) and more complex (RENAL score of 6 vs. 5; *p* < 0.001). Operative time was longer in the RAPN group (143 vs. 122 min; *p* < 0.01), while warm ischaemia time did not differ significantly (16 vs. 15 min; *p* = 0.37). Estimated blood loss was lower (0 vs. 10 mL; *p* = 0.049) and the hospital stay was shorter (3 vs. 4 days; *p* < 0.001) in the RAPN group. Haemoglobin decrease and postoperative creatinine change were comparable between groups. Positive surgical margins were observed less frequently in the RAPN group (2.3% vs. 7.7%), but this difference was not statistically significant (*p* = 0.34). Complication rates were significantly lower in the RAPN group (4% vs. 22%; *p* < 0.05), with no major complications observed in the robotic cohort. **Conclusions:** In this institutional experience, RAPN was associated with favourable perioperative outcomes during the transition period, despite the treatment of larger and more complex renal tumours. The slightly longer operative and warm ischaemia times likely reflect a more comprehensive reconstruction strategy, which may contribute to improved haemostatic control and lower complication rates. Further studies with extended follow-up are required to evaluate oncological and renal functional outcomes.

## 1. Introduction

Partial nephrectomy (PN) has become the standard surgical approach for the management of localized renal tumours, particularly for clinical T1 lesions, whenever technically feasible [1]. The primary objective of nephron-sparing surgery is to achieve optimal oncological control while preserving renal function and minimizing perioperative morbidity [2]. The preservation of functional renal parenchyma is especially important in reducing the risk of chronic kidney disease and its associated cardiovascular consequences [1].

Over the past two decades, minimally invasive approaches have progressively replaced open surgery in selected patients. Laparoscopic partial nephrectomy (LPN) represented the first major step toward minimally invasive nephron-sparing surgery, offering reduced postoperative pain, shorter hospital stays and faster recovery compared with open surgery [3]. However, despite these advantages, LPN remains technically demanding, particularly during tumour excision and intracorporeal reconstruction. Precise tumour resection, haemostasis and renorrhaphy must often be performed under time constraints imposed by warm ischaemia, which may impact perioperative and functional outcomes [4].

The introduction of robotic surgery has significantly changed the landscape of minimally invasive urology. Robot-assisted partial nephrectomy (RAPN) offers enhanced three-dimensional visualization, improved ergonomics and articulated instruments that facilitate precise dissection and suturing [5,6,7]. These technical advantages are particularly relevant during the reconstructive phase, where the accurate closure of the collecting system and renal parenchyma is essential to minimize complications such as bleeding and urinary leakage [6,7].

Several comparative studies and meta-analyses have demonstrated that RAPN is associated with reduced blood loss, lower complication rates and improved perioperative outcomes compared with LPN, while maintaining equivalent oncological efficacy [8,9,10]. Furthermore, robotic assistance has been shown to expand the indications for nephron-sparing surgery, enabling safe treatment of larger and more complex tumours [11].

An important aspect of partial nephrectomy is the management of warm ischaemia. While shorter ischaemia times are traditionally considered beneficial for renal function preservation [4], recent evidence suggests that the quality of tumour resection and reconstruction, including meticulous closure of the collecting system and parenchyma, may be equally important in preventing complications and ensuring optimal outcomes [12,13]. In robotic surgery, surgeons may deliberately accept slightly longer warm ischaemia times to achieve more precise and secure reconstruction [12,13].

Despite the growing body of evidence supporting RAPN, data describing the real-world institutional transition from laparoscopic to robotic partial nephrectomy remain limited [14,15]. Such transition periods are clinically relevant, as they reflect changes in the surgical strategy, case selection and technical approach [14,15].

At our institution, laparoscopic partial nephrectomy was the standard minimally invasive technique for many years, with more than 200 procedures performed. Following the introduction of robotic surgery, RAPN progressively replaced LPN and became the standard approach. Notably, this transition was accompanied by a shift in surgical strategy, including more meticulous reconstruction techniques, such as the closure of both the tumour bed and renal parenchyma.

The aim of this study was therefore to compare perioperative outcomes, surgical margin status and early functional outcomes between the last 50 laparoscopic partial nephrectomies and the first 50 robot-assisted partial nephrectomies performed at our centre. We hypothesized that RAPN would enable the treatment of more complex tumours while maintaining favourable perioperative and surgical outcomes, despite potentially longer warm ischaemia time due to more comprehensive renal reconstruction.

## 2. Materials and Methods

### 2.1. Study Design and Patient Population

This study was designed as a retrospective, single-centre cohort analysis conducted at the Department of Urology, University Medical Centre Ljubljana, Slovenia. A total of 100 consecutive patients undergoing minimally invasive partial nephrectomy were included. Two cohorts were defined based on the surgical approach and time period:

- The last 50 laparoscopic partial nephrectomies (LPN) performed between August 2014 and May 2018.
- The first 50 robot-assisted partial nephrectomies (RAPN) performed between June 2018 and February 2020.

The temporal sequence reflects the institutional adoption of robotic surgery in 2018, after which robot-assisted partial nephrectomy progressively replaced laparoscopic partial nephrectomy as the standard minimally invasive approach.

Patients were eligible if they underwent partial nephrectomy for a radiologically confirmed renal mass with curative intent. Patients undergoing radical nephrectomy, ablative procedures or those with incomplete key perioperative data were excluded.

All procedures were performed consecutively during the study period, reflecting real-world institutional practice and reducing potential selection bias.

### 2.2. Preoperative Evaluation

All patients underwent standard preoperative assessment, including medical history, physical examination, laboratory testing and cross-sectional imaging. Tumour characteristics were assessed using computed tomography or magnetic resonance imaging. Tumour complexity was evaluated using the RENAL nephrometry score, which was available for 49 patients in the LPN group and 47 patients in the RAPN group.

Baseline variables included age, sex, body mass index, tumour size, tumour location, RENAL nephrometry score and preoperative renal function. Renal function was assessed using estimated glomerular filtration rate (eGFR).

### 2.3. Surgical Technique

All procedures were performed by three experienced minimally invasive urologic surgeons. The surgical approach reflected the institutional standard at the time of surgery.

#### 2.3.1. Laparoscopic Partial Nephrectomy (LPN)

Laparoscopic partial nephrectomy was performed using a standard transperitoneal approach. After mobilization of the colon, the kidney was exposed and the renal hilum was identified and dissected. In most cases, arterial clamping was performed without routine venous clamping. Off-clamp procedures were performed selectively in anatomically favourable tumours.

Tumour excision was carried out using cold scissors with an attempt to preserve healthy renal parenchyma. Reconstruction in the laparoscopic cohort was primarily limited to parenchymal suturing, focusing on haemostasis of the resection bed. Closure of the collecting system or tumour bed was performed selectively.

#### 2.3.2. Robot-Assisted Partial Nephrectomy (RAPN)

Robot-assisted partial nephrectomy (RAPN) was performed using the da Vinci Xi surgical system (Intuitive Surgical, Sunnyvale, CA, USA) via a transabdominal approach, following the technique previously described by Cerović and Hawlina [16].

Patients were positioned in a modified flank position. After establishment of pneumoperitoneum, a transperitoneal approach was used with standard trocar placement (Figure S1). The colon was mobilized to expose the kidney, and the renal hilum was carefully dissected.

Hilar control was achieved using clamping of the renal artery, while the renal vein was routinely left unclamped. This approach was intended to preserve venous outflow during tumour excision. Tumour excision was carried out under warm ischaemia using cold scissors, aiming for complete tumour removal with preservation of surrounding renal parenchyma.

Whenever anatomically feasible, tumour excision was performed using an enucleation-based technique, following the natural plane between the tumour pseudocapsule and the surrounding renal parenchyma.

Selective arterial clamping and intraoperative ultrasound or indocyanine green fluorescence were used at the surgeon’s discretion. Off-clamp procedures were rarely performed and were reserved for selected highly exophytic, low-complexity tumours where adequate visualisation and haemostasis were anticipated without hilar clamping. Selective arterial clamping was used when preoperative imaging identified a tumour-specific arterial branch that could be safely isolated. In such cases, indocyanine green (ICG) fluorescence was used to confirm selective renal devascularisation. Intraoperative ultrasound was primarily used for endophytic tumours or when tumour depth and margins were not clearly defined on preoperative imaging, whereas it was generally not required for highly exophytic and clearly visible lesions.

Reconstruction was performed in a two-layer fashion. First, the tumour bed and collecting system, when entered, were closed using a continuous suture. This was followed by parenchymal renorrhaphy using sliding-clip or interrupted sutures to achieve haemostasis and restore renal contour.

#### 2.3.3. Key Technical Differences Between Approaches

Several important technical differences between the laparoscopic and robotic approaches were observed.

First, tumour excision strategy differed between the cohorts. In the laparoscopic cohort, resection was typically performed with a small margin of normal parenchyma. In contrast, the robotic approach more frequently enabled tumour enucleation along the natural pseudocapsular plane, allowing for maximal preservation of renal tissue.

Second, reconstructive strategy evolved following the adoption of robotic surgery. While laparoscopic procedures primarily relied on parenchymal suturing, robotic procedures more consistently involved systematic closure of the tumour bed and collecting system, followed by parenchymal renorrhaphy.

These technical differences likely reflect both the advantages of the robotic platform and the institutional evolution of surgical technique over time.

### 2.4. Outcome Measures

The primary and secondary outcome measures were predefined and included demographic, tumour-related, perioperative, early functional and pathological parameters. Baseline characteristics included patient age, tumour size and tumour complexity, assessed using the RENAL nephrometry score.

Perioperative outcomes comprised operative time, warm ischaemia time, estimated blood loss and length of hospital stay. Early functional outcomes were evaluated by comparing preoperative and postoperative estimated glomerular filtration rate (eGFR) and haemoglobin values, with particular emphasis on perioperative changes in these parameters. Postoperative laboratory values, including haemoglobin concentration and eGFR, were routinely assessed within 48–72 h after surgery.

Pathological outcomes included histopathological findings and surgical margin status, classified as R0 or R1 resection. In cases of synchronous tumours, only the dominant lesion was considered for histopathological analysis.

Postoperative complications were recorded and graded according to the Clavien–Dindo classification, with particular attention to major complications defined as Clavien–Dindo grade III or higher.

### 2.5. Statistical Analysis

Continuous variables were expressed as median (interquartile range, IQR) and categorical variables as frequencies and percentages.

Comparisons between groups were performed using the Mann–Whitney U test for continuous variables and the chi-square or Fisher’s exact test for categorical variables, as appropriate.

A *p*-value < 0.05 was considered statistically significant. Statistical analysis was performed using IBM SPSS Statistics, Version 29.0 (IBM Corp., Armonk, NY, USA).

## 3. Results

### 3.1. Patient Characteristics

A total of 100 patients were included in the analysis, comprising 50 patients who underwent laparoscopic partial nephrectomy (LPN) and 50 patients who underwent robot-assisted partial nephrectomy (RAPN).

The baseline characteristics are summarized in Table 1. There was no significant difference in age between the groups (64.5 vs. 63 years, *p* = 0.56). Similarly, no significant differences were observed in sex distribution (male sex: 62% vs. 68%, *p* = 0.36), ASA score (2 vs. 2, *p* = 0.63), or tumour side (left-sided tumours: 46% vs. 60%, *p* = 0.15).

Tumours in the RAPN group were significantly larger (3.4 vs. 2.5 cm, *p* = 0.031) and more complex, as reflected by higher RENAL nephrometry scores (6 vs. 5, *p* < 0.001). Anterior tumour location was more frequently observed in the RAPN group, although the difference did not reach statistical significance (56.5% vs. 39.1%, *p* = 0.10).

### 3.2. Perioperative Outcomes

Perioperative and early functional outcomes are presented in Table 2.

Operative time was significantly longer in the RAPN group (143 vs. 122 min, *p* < 0.01). Warm ischaemia time did not differ significantly between groups (16 vs. 15 min, *p* = 0.37), while only one case in each group was performed without hilar clamping.

Estimated blood loss was lower in the RAPN group (0 vs. 10 mL, *p* = 0.049), and the length of hospital stay was significantly shorter (3 vs. 4 days, *p* < 0.001).

The decrease in haemoglobin levels was comparable between groups (20 vs. 22 g/L, *p* = 0.56). Similarly, early postoperative eGFR decline was comparable between groups (−1 vs. 0 mL/min/1.73 m^2^, *p* = 0.256).

### 3.3. Pathological Outcomes and Perioperative Complications

Pathological outcomes and perioperative complications are summarized in Table 3.

Positive surgical margins were analysed only in patients with malignant histopathological findings. Positive surgical martins were identified in 3/39 patients in the LPN group and 1/44 in the RAPN group, without a statistically significant difference between the groups (*p* = 0.34).

Postoperative complications occurred in eleven patients (22%) in the LPN group and two patients (4%) in the RAPN group (*p* = 0.013).

In the laparoscopic cohort, four major complications (Clavien–Dindo ≥ III) were observed, including postoperative bleeding requiring selective arterial embolization, arteriovenous fistula and cases of hemorrhagic shock.

In contrast, only minor complications (Clavien–Dindo I) were observed in the RAPN group, including transient hematuria and a small port-site hernia. No major complications were recorded in the robotic cohort.

In cases of synchronous tumours, only the dominant lesion was considered for classification.

Histopathological distribution was comparable between groups (*p* = 0.17), with clear cell renal cell carcinoma being the most common subtype in both groups, accounting for 60% of tumours in the LPN group and 56% in the RAPN group.

Papillary renal cell carcinoma was more frequently observed in the RAPN group (28% vs. 12%), whereas benign lesions were more common in the LPN group (22% vs. 12%).

Other renal cell carcinoma subtypes were rare in both cohorts.

## 4. Discussion

In this study, we evaluated the outcomes of an institutional transition from laparoscopic to robot-assisted partial nephrectomy in a high-volume European centre. Our results indicate that robot-assisted partial nephrectomy (RAPN) is associated with favourable perioperative outcomes and non-inferior surgical margin rates, despite being performed on patients with significantly larger and more complex tumours.

Partial nephrectomy is currently considered the standard of care for localized renal tumours, as recommended by international guidelines, due to its ability to preserve renal function while maintaining oncological control [1,2]. Minimally invasive approaches have progressively replaced open surgery, with laparoscopic partial nephrectomy (LPN) representing an important milestone in the evolution of nephron-sparing surgery [3]. However, LPN remains technically demanding, particularly during tumour excision and intracorporeal suturing [17].

During the laparoscopic era at our institution, some highly complex renal masses were preferentially managed with open partial nephrectomy. Following the introduction of robotic surgery, increasingly complex tumours were more frequently approached using minimally invasive nephron-sparing techniques. This may reflect the higher tumour complexity observed in the RAPN cohort.

The introduction of robotic surgery has addressed many of these technical limitations. RAPN offers improved visualization, ergonomics and dexterity, facilitating complex tumour resections and renal reconstruction [5,6]. In this present study, RAPN was used in significantly larger and more complex tumours, as reflected by higher RENAL nephrometry scores [18], which is consistent with previous reports demonstrating the expansion of minimally invasive nephron-sparing surgery toward more challenging renal masses [6,19].

Estimated blood loss was significantly lower in the RAPN group, which is consistent with previous comparative studies and meta-analyses [20,21,22]. Improved visualization and more precise vascular control may have contributed to this finding. Despite this, the decrease in haemoglobin levels was comparable between groups, which may reflect perioperative haemodilution rather than true differences in blood loss.

One of the most clinically relevant findings of this study is the significantly lower complication rate in the robotic cohort. While LPN was associated with a considerable proportion of major complications, including postoperative bleeding requiring embolization and cases of hemorrhagic shock, no major complications were observed in the RAPN group. These findings are consistent with previous reports demonstrating lower morbidity following robotic surgery [8,9].

Several technical factors may explain these differences. First, the robotic platform allows for more precise identification of anatomical planes and improved vascular control. Second, tumour enucleation was more frequently performed in the robotic cohort, following the natural pseudocapsular plane. This technique has been shown to preserve renal parenchyma and reduce bleeding while maintaining oncological safety [23,24].

Third, reconstructive strategy differed significantly between the approaches. In the laparoscopic cohort, reconstruction was primarily limited to parenchymal suturing, whereas RAPN enabled a more comprehensive two-layer closure of the tumour bed and renal parenchyma. This approach may contribute to haemostatic control and may be associated with reduced complications such as postoperative bleeding and urinary leakage.

Warm ischaemia time was slightly longer in the robotic group, although the difference was not statistically significant. Traditionally, minimizing ischaemia time has been considered important for renal function preservation [4]. However, more recent evidence suggests that within clinically acceptable limits, the quality of tumour excision and reconstruction may be equally important [12,13,25]. In our cohort, the slightly longer ischaemia time likely reflects a more meticulous reconstructive strategy rather than a technical limitation.

Another important finding is the numerically lower rate of positive surgical margins in the robotic group, although this difference did not reach statistical significance (*p* = 0.34). This observation is in line with previous studies, which have also reported low and comparable positive margin rates after RAPN without statistically significant differences between surgical approaches [8,26].

The histopathological distribution observed in our study reflects real-world clinical practice, with clear cell renal cell carcinoma being the most common subtype. The presence of benign lesions and less common histological subtypes further highlights the heterogeneity of renal masses encountered in contemporary nephron-sparing surgery.

An important consideration is the effect of surgical experience. Since the completion of the study period, our institutional experience with RAPN has exceeded 280 procedures. With increasing experience, operative time and warm ischaemia time have likely decreased, reflecting the well-described learning curve associated with robotic surgery [5,15,27]. Therefore, the outcomes reported in this study may underestimate the contemporary performance of RAPN in our centre.

The strengths of this study include its real-world design and the evaluation of a complete institutional transition from laparoscopic to robotic surgery. This design provides valuable insight into how surgical practice evolves in a high-volume centre.

However, several limitations must be acknowledged. The retrospective design is associated with potential selection bias. The sequential nature of the cohorts may reflect temporal improvements in surgical technique, perioperative care and institutional experience over time. The relatively small sample size limits statistical power, while long-term oncological follow-up and renal function outcomes were not assessed.

Importantly, this present study reflects not only a transition between surgical platforms but also an institutional evolution in minimally invasive nephron-sparing surgery. During the laparoscopic era, some highly complex renal tumours were preferentially managed with open partial nephrectomy, whereas the introduction of robotic surgery enabled the minimally invasive treatment of increasingly complex renal masses. In parallel, surgical strategy evolved over time, including more frequent tumour enucleation, more comprehensive reconstructive techniques and changes in perioperative management. Therefore, the observed differences between cohorts likely reflect a combination of robotic platform advantages, increasing surgical experience, technical evolution and institutional learning curve effects.

## 5. Conclusions

The transition from laparoscopic to robot-assisted partial nephrectomy in our institution was associated with favourable perioperative outcomes during the transition period despite the treatment of larger and more complex renal tumours. The slightly longer warm ischaemia time observed during the early robotic experience likely reflects a more comprehensive reconstruction strategy, which may have contributed to the observed reduction in complication rates. These findings highlight the evolving role of robotic surgery and surgical experience in contemporary minimally invasive nephron-sparing surgery.

## Figures and Tables

**Table 1:** Baseline characteristics.

Variable	LPN (*n* = 50)	RAPN (*n* = 50)	*p*-Value
Age (years)	64.5 (57.5–71)	63 (54–71)	0.56
Tumour size (cm)	2.5 (2–3.5)	3.4 (2.5–4.0)	0.031
RENAL score	5 (4–6)	6 (5–7)	<0.001
Sex, male %	62	68	0.36
ASA score	2 (2–3)	2 (2–3)	0.63
Tumour side, left %	46	60	0.15
Anterior or posterior location, anterior %	39.1	56.5	0.10

**Table 2:** Perioperative and early functional outcomes.

Variable	LPN (*n* = 50)	RAPN (*n* = 50)	*p*-Value
Operative time (min)	122 (91–150)	143 (114–172)	<0.01
Warm ischaemia time (min)	15 (12–18)	16 (13.8–20.2)	0.37
Estimated blood loss (mL)	10 (10–50)	0 (0–50)	0.049
Length of stay (days)	4 (3–6)	3 (3–3)	<0.001
Haemoglobin drop (g/L)	20 (13–28)	22 (15–26)	0.56
Early eGF change (mL/min/1.73 m^2^)	−1 (−8.8–0)	0 (−2–1)	0.256

**Table 3:** Pathological outcomes and perioperative complications.

Variable	LPN (*n* = 50)	RAPN (*n* = 50)	*p*-Value
Positive margins (R1) (n, %)	3 (7.7%), n = 39	1 (2.3%), n = 44	0.34
Overall complication (n, %)	11 (22%)	2 (4%)	0.013
C–D ≥ 3 complication (n, %)	4 (8%)	0 (0%)	0.12
Histology			0.17
ccRCC (n, %)	30 (60%)	28 (56%)	
pRCC (n, %)	6 (12%)	14 (28%)	
Other RCC (n, %)	3 (6%)	2 (4%)	
Benign tumours (n, %)	11 (22%)	6 (12%)	

C–D ≥ 3—Clavien–Dindo grade complications more than 3, ccRCC—clear cell renal cell carcinoma, pRCC—papillary renal cell carcinoma.

## Data Availability

The data supporting the findings of this study are not publicly available due to privacy and ethical restrictions but are available from the corresponding author upon reasonable request.

## Supplementary Materials

The following supporting information can be downloaded at: https://www.mdpi.com/article/10.3390/jcm15124746/s1, Figure S1: Port placement for robot-assisted partial nephrectomy for right side. Green—8 mm robotic ports, blue—12 mm assistant port, orange—5 mm liver retractor port.

## Abbreviations

The following abbreviations are used in this manuscript:

PN	Partial nephrectomy
LPN	Laparoscopic partial nephrectomy
RAPN	Robot-assisted partial nephrectomy
RCC	Renal cell carcinoma
eGF	Estimated glumerular filtration
