# Supplementary material for: Transition from Laparoscopic to Robot-Assisted Partial Nephrectomy: Perioperative Outcomes During an Institutional Transition in a High-Volume European Centre
Source: J Clin Med. 2026 Jun 18;15(12):4746. doi: 10.3390/jcm15124746 (PMC13302577; doi:10.3390/jcm15124746)
Supplement: Supplementary file 1 [file jcm-15-04746-s001.zip › jcm-4335864-supplementary.pdf]

Supplementary material

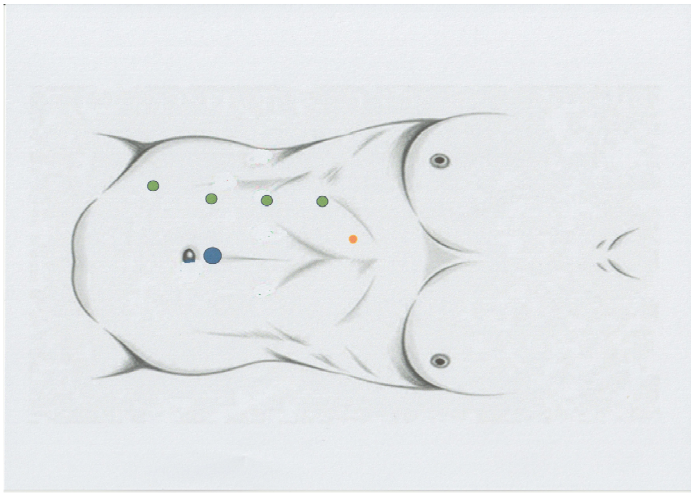

Figure S1. Port placement for robot-assisted partial nephrectomy for right side. Green—8 mm robotic ports, blue—12 mm assistant port, orange—5 mm liver retractor port.
